# Characterization of a feruloyl esterase from *Aspergillus terreus* facilitates the division of fungal enzymes from Carbohydrate Esterase family 1 of the carbohydrate‐active enzymes (CAZy) database

**DOI:** 10.1111/1751-7915.13273

**Published:** 2018-04-26

**Authors:** Miia R. Mäkelä, Adiphol Dilokpimol, Salla M. Koskela, Jaana Kuuskeri, Ronald P. de Vries, Kristiina Hildén

**Affiliations:** ^1^ Department of Microbiology Faculty of Agriculture and Forestry University of Helsinki Viikinkaari 9 Helsinki Finland; ^2^ Fungal Physiology Westerdijk Fungal Biodiversity Institute & Fungal Molecular Physiology Utrecht University Uppsalalaan 8 3584 CT Utrecht The Netherlands; ^3^ Present address: School of Biotechnology Royal Institute of Technology Alba Nova University Centre Roslagstullsbacken 21 Stockholm Sweden

## Abstract

Feruloyl esterases (FAEs) are accessory enzymes for plant biomass degradation, which catalyse hydrolysis of carboxylic ester linkages between hydroxycinnamic acids and plant cell‐wall carbohydrates. They are a diverse group of enzymes evolved from, e.g. acetyl xylan esterases (AXEs), lipases and tannases, thus complicating their classification and prediction of function by sequence similarity. Recently, an increasing number of fungal FAEs have been biochemically characterized, owing to their potential in various biotechnological applications and multitude of candidate FAEs in fungal genomes. However, only part of the fungal FAEs are included in Carbohydrate Esterase family 1 (CE1) of the carbohydrate‐active enzymes (CAZy) database. In this work, we performed a phylogenetic analysis that divided the fungal members of CE1 into five subfamilies of which three contained characterized enzymes with conserved activities. Conservation within one of the subfamilies was confirmed by characterization of an additional CE1 enzyme from *Aspergillus terreus*. Recombinant *A. terreus* FaeD (AtFaeD) showed broad specificity towards synthetic methyl and ethyl esters, and released ferulic acid from plant biomass substrates, demonstrating its true FAE activity and interesting features as potential biocatalyst. The subfamily division of the fungal CE1 members enables more efficient selection of candidate enzymes for biotechnological processes.

## Introduction

Plant biomass is a vast resource of renewable compounds that are essential for the development of a sustainable bio‐based economy. In cell walls of gramineous plants, hemicelluloses are cross‐linked to the aromatic lignin polymer via hydroxycinnamic acids (ferulic acid and *p*‐coumaric acid) or through (4‐*O*‐methyl)glucuronic acid (Kroon *et al*., 1999; Harris, 2005). Feruloyl esterases [ferulic acid esterases (FAEs), EC 3.1.1.73] are enzymes that catalyse the cleavage of covalent ester linkage between a phenolic acid and poly‐ or oligosaccharide liberating hydroxycinnamic acids from plant biomass (Wong, 2006; Faulds, 2010). FAEs act as accessory enzymes enabling other enzymes, such as xylanases and pectinases, to gain access to their specific substrates in the plant cell wall. Due to their ability to hydrolyse ester linkages and perform transesterification reactions, FAEs are promising biocatalysts for a broad range of biotechnological applications. These include pharmaceutical, agricultural and food industries, as well as production of biofuels and biochemicals (reviewed in Dilokpimol *et al*. (2016)).

FAEs are a highly diverse group of plant biomass active enzymes that have evolved from various type of enzymes, such as acetyl xylan esterases (AXEs), lipases and tannases (Benoit *et al*., 2008). This may explain notable differences in the specificity of FAEs, e.g. towards different hydroxycinnamate model substrates (Dilokpimol *et al*., 2017; Faulds *et al*., 2005; Kroon *et al*., 2000). The increasing number of fungal genome sequences has offered a multitude of putative FAE sequences, and resulted in an increasing number of biochemically characterized FAEs (Dilokpimol *et al*., 2018). Recently, we presented an updated classification of fungal FAEs based on evolutionary relationships among fungal FAE sequences and their orthologs from a set of genome sequences (Dilokpimol *et al*., 2016). As a result, fungal FAEs were divided into 13 subfamilies (SFs), which was discussed with respect to their substrate specificity.

However, only some fungal FAEs from this phylogeny‐based classification, namely those present in SF5 and SF6 (Dilokpimol *et al*., 2016), are catalogued in Carbohydrate Esterase family 1 (CE1) of the carbohydrate‐active enzymes (CAZy) database (Lombard *et al*., 2014). CE1 has a long history and was first described as a family of AXEs in fungi, while later also fungal FAEs were described for this family (Coutinho and Henrissat, 1999). In contrast, the characterized bacterial enzymes of the family CE1 possess various activities ranging from FAEs to cellulose acetate esterases and antigen 85 proteins with diacylglycerol acyltransferase/mycolyltransferase activity (http://www.cazy.org).

As the fungal CE1 enzymes characterized so far have been shown to include both AXEs and FAEs, we explored whether these activities are present as distinct subfamilies of CE1 using phylogenetic analysis. This analysis was facilitated by an initial biochemical characterization of several other CE1 enzymes (Dilokpimol *et al*., 2018) as well as by the more detailed characterization of an additional CE1 enzyme from *Aspergillus terreus* as a part of this study.

## Results and discussion

### Phylogenetic analysis reveals five subfamilies in fungal part of CE1

**Figure 1:**
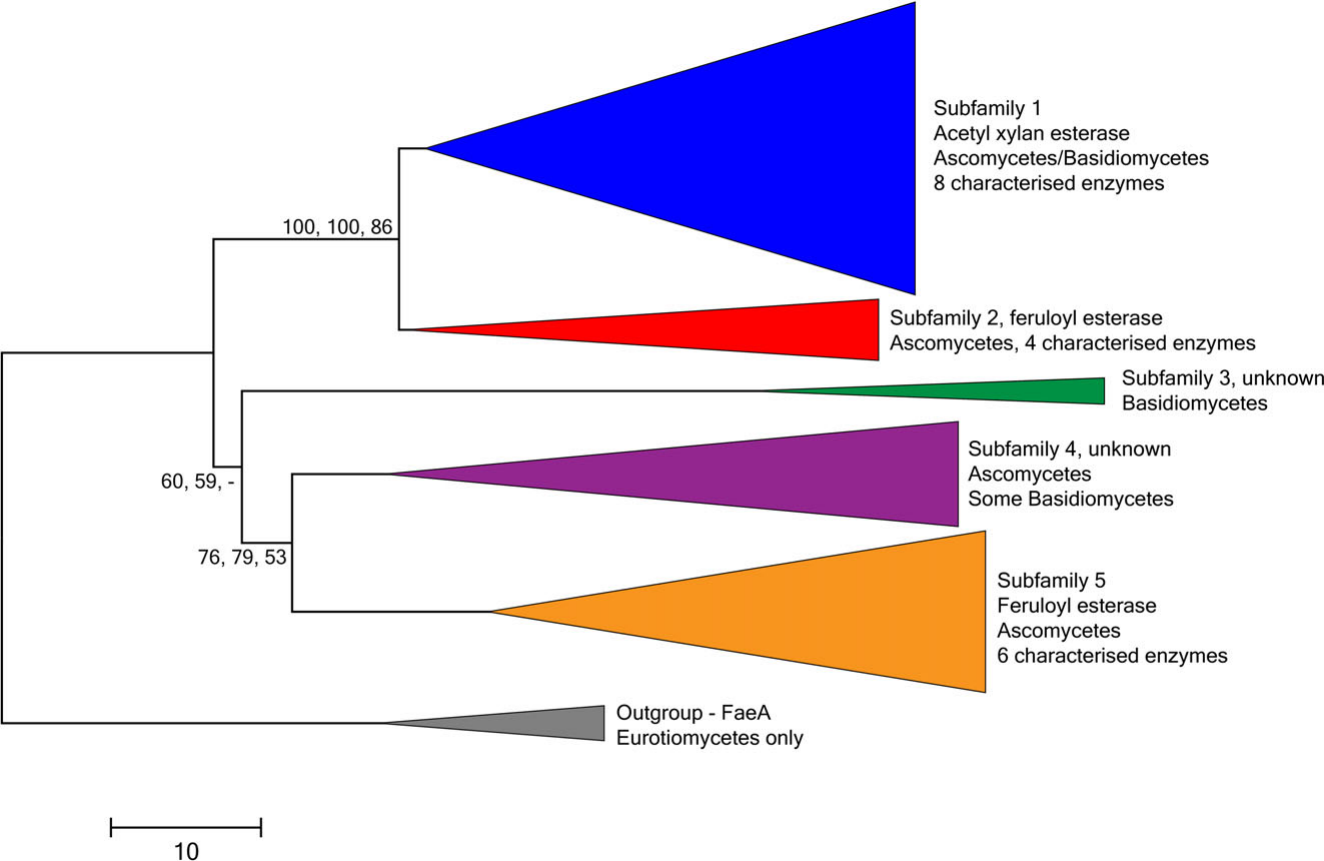
Phylogenetic tree of fungal CE1 genes based on amino acid sequences. The evolutionary history was inferred using the neighbour‐joining (NJ) method (Saitou and Nei, 1987) and the optimal tree is shown, with the main branches collapsed. Bootstrap values (500 replicates) > 50 are shown next to the branches (Felsenstein, 1985), also of a minimal evolution (ME) and maximum likelihood (ML) tree using the same dataset (order: NJ, ME, ML). The tree is drawn to scale, with branch lengths in the same units as those of the evolutionary distances used to infer the phylogenetic tree. Evolutionary analyses were conducted in MEGA7 (Kumar *et al*., 2016). Eight FAE SF7 (Dilokpimol *et al*., 2016) sequences were used as an outgroup. The function of each branch is indicated where possible, as well as the fungal orders it contains and the number of functionally characterized enzymes. The full phylogenetic tree can be found as Fig. S1.

Considering the highly diverse functions and differences in gene length of fungal and bacterial CE1 members (http://www.cazy.org), we focused on the fungal esterases in this study. So far, all characterized fungal CE1 enzymes are either AXEs or FAEs and we therefore studied whether these activities can be distinguished as different subfamilies of CE1. In total > 200 CE1 amino acid sequences were obtained from JGI Mycocosm, and after manually filtering these for gene model errors and recent duplications, 148 CE1 amino acid sequences were used for the final alignment (Table S1) and phylogeny (Fig. S1). *Aspergillus niger* FaeA and seven of its orthologs were used as an outgroup. NJ, ME and ML bootstrap trees were generated, which revealed the presence of five subfamilies in this set of fungal CE1 genes (Figs 1 and S1).

Fungal CE1 subfamily 1 contains eight biochemically characterized enzymes, which are present in all but two of the branches of this subfamily (Figs 1 and S1). All characterized subfamily 1 enzymes are AXEs from both asco‐ and basidiomycete species, suggesting that this whole subfamily consists of enzymes with AXE activity. They include five AXEs from *Aspergillus* species, i.e., *A. awamori* AXE (Koseki *et al*., 1997), *A. niger* AxeA (de Graaff *et al*., 1992), *A. ficuum* AXE (Chung *et al*., 2002), *A. oryzae* AxeA (Koseki *et al*., 2006) and *A. nidulans* AXE (Bauer *et al*., 2006), which cluster together with other candidate enzymes from Eurotiomycetes, *Myceliophthora thermophila* Axe3 (Pouvreau *et al*., 2011), *Volvariella volvacea* AxeI/Axe1 (Ding *et al*., 2007) and *Phanerochaete chrysosporium* AXE (Huy *et al*., 2013). The two subfamily 1 branches containing no characterized enzymes include candidates from diverse asco‐ and basidiomycete species from the clades Dothidiomycetes, Leotiomycetes, Sordariomycetes and Agaricomycotina (Figs 1 and S1). Characterization of enzymes from these two subfamily 1 branches would be interesting to confirm if they also are AXEs.

Two FAE‐specific subfamilies, 2 and 5, were distinguished in the phylogenetic tree, and they both contain only ascomycete sequences (Figs 1 and S1). Subfamily 2 includes four characterized enzymes, *Aspergillus sydowii* AsFaeE (Dilokpimol *et al*., 2018), *Talaromyces funiculosus* cinnamoyl esterase (Kroon *et al*., 2000), *Neurospora crassa* FaeB (Crepin *et al*., 2003) and *M. thermophila* FAE (Dilokpimol *et al*., 2018), all of which have been shown to possess activity towards synthetic methyl esters. Their presence in all branches of subfamily 2 suggests that this is a FAE subfamily. An esterase hydrolysing both artificial cinnamic and benzoic acid esters, but not complex natural FAE substrates, i.e. feruloylated saccharides, arabinoxylan, sugar beet pectin (SBP) and de‐starched wheat bran, has been described from the basidiomycete fungus *Auricularia auricularia‐judae* (Haase‐Aschoff *et al*., 2013), but since the gene has not been identified it cannot be assigned to a FAE family at this point. This shows the high diversity of fungal esterases, and therefore, the CE1 subfamily 2 enzymes should be tested on natural substrates to confirm their true FAE activity.

Six characterized FAEs are present in both main branches of subfamily 5 of CE1 (Figs 1 and S1). These include *N. crassa* FaeD (Crepin *et al*., 2004), *A. sydowii* FaeC and FaeD1 (Dilokpimol *et al*., 2018), *A. nidulans* FaeC (Debeire *et al*., 2012) and *A. niger* FaeC (Dilokpimol *et al*., 2017), as well as AtFaeD characterized in this study, thus supporting that CE1 subfamily 5 is also a FAE subfamily. In addition to the artificial FAE substrates, several of the functionally characterized subfamily 5 enzymes have also been shown to release ferulic acid from natural plant biomass substrates, such as wheat arabinoxylan (WAX) and SBP (Crepin *et al*., 2003; Dilokpimol *et al*., 2017), or hydrolyse feruloylated oligosaccharides (Debeire *et al*., 2012).

For two of the five CE1 subfamilies, subfamilies 3 and 4, no characterized members are available and therefore no function can be assigned to them at this point (Figs 1 and S1). The small subfamily 3 consists only of basidiomycete sequences, while subfamily 4 contains mainly ascomycete sequences and only a few basidiomycete sequences. Analysis of some enzymes of these subfamilies would be needed to determine whether they are AXEs or FAEs or possess another activity.

### *Aspergillus terreus* FaeD hydrolyses a broad range of synthetic ester‐linkage‐containing substrates

As an example case to confirm the consistency of the substrate specificity within the subfamilies of CE1, we heterologously expressed and characterized *A. terreus* FaeD. This enzyme is a member of subfamily 5 (Figs 1 and S1), which also contains the recently characterized AnFaeC from *A. niger* (Dilokpimol *et al*., 2017) in a different branch. The Aspergilli appear to have abundant numbers of putative FAE‐encoding genes, and previously clear differences have been reported between the three characterized FAEs from *A. niger* (de Vries *et al*., 2002; Dilokpimol *et al*., 2017).

AtFaeD from *A. terreus* was successfully produced in the fed‐batch fermentation in *Pichia pastoris* as active 43 kDa protein (Fig. S2A–C), which is higher than the theoretical molecular mass of the mature protein sequence, 26.8 kDa. At least seven putative *N*‐glycosylation and two putative *O*‐glycosylation sites were predicted in the AtFaeD amino acid sequence, some of which are quite close to each other, suggesting that the recombinant protein could be hyperglycosylated. However, the molecular mass of the recombinant AtFaeD did not change after treatment with the glycosidase enzymes, PNGase F, Endo H_f_ or *O*‐glycosidase (Fig. S2B). Also, no protein bands corresponding to the monomeric AtFaeD peptide were detected under harsh reducing conditions in SDS‐PAGE, suggesting that the protein was not a dimer. Because the treatment with the glycosidases did not change the molecular mass of AtFaeD, we analysed the carbohydrate content of the sample using phenol‐sulphuric acid assay (Masuko *et al*., 2005). This showed a high carbohydrate content, 2 mg ml^−1^, compared with the concentration of AtFaeD (0.84 mg ml^−1^), which may be due to the fact that the AtFaeD was not purified and the sample still contained other glycosylated *P. pastoris* extracellular proteins. Considering that the AtFaeD is the major protein present in the sample (Fig. S2), it is possible that the AtFaeD is hyperglycosylated, but the conformation of the protein or the attached oligosaccharide chains may prevent the glycosidases from hydrolysing it. However, the presence of this 43 kDa band only in the *P. pastoris* transformant that expresses the AtFaeD‐encoding gene, as well as the response to the anti‐His antibody, leaves no doubt that this protein is in fact AtFaeD. The theoretical isoelectric point of AtFaeD was 4.34.

**Figure 2:**
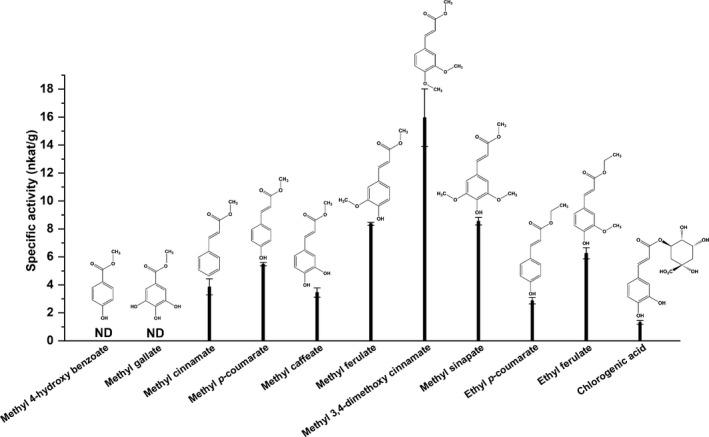
Specific activity of AtFaeD towards different synthetic methyl and ethyl esters, and chlorogenic acid. Vertical error bars represent standard deviation of three replicate measurements. ND = not detected.

Recombinant AtFaeD displayed broad substrate specificity and was active towards all tested synthetic ester linkage containing monomeric substrates, except methyl 4‐hydroxybenzoate (Fig. 2). This supports the phylogenetic placement of AtFaeD in CE1 subfamily 5 together with *A. niger* AnFaeC (Dilokpimol *et al*., 2017) and *N. crassa* NcFaeD (Crepin *et al*., 2004), which also have a broad substrate range (Fig. S1). Furthermore, these enzymes are classified into SF5 in the recent phylogenetic classification of fungal FAEs (Dilokpimol *et al*., 2016) that includes additional broad substrate FAEs, such as *Aspergillus clavatus* AcFAE (Damásio *et al*., 2013) and *M. thermophila* ClFaeA1 (Kühnel *et al*., 2012). Similar to AnFaeC (Dilokpimol *et al*., 2017) and *Talaromyces stipitatus* FaeC (Vafiadi *et al*., 2006), AtFaeD was not active on methyl 4‐hydroxybenzoate that has only one carbon in the aliphatic side‐chain. This is in line with previous suggestions that a correct distance between the aromatic group and the ester bond is necessary for the catalytic activity of FAEs (Kroon *et al*., 1997; Topakas *et al*., 2005). Furthermore, AtFaeD showed higher activity on methoxy‐substituted model compounds than on their hydroxylated counterparts, as well as on the compounds containing two methoxy substitutions on the phenyl ring (Fig. 2). It preferred methyl esters to ethyl esters.

The highest activity of AtFaeD, 12 nkat/g (720 mU g^−1^), was detected with methyl 3,4‐dimethoxycinnamate, bearing C‐3 and C‐4 methoxy substitutions (Fig. 2). Approximately 50% lower activity was measured towards methyl ferulate and methyl sinapate as substrates. AtFaeD displayed low activity, 1 nkat/g (60 mU g^−1^), towards chlorogenic acid, a compound present in several agricultural by‐products, such as coffee pulp and apple marc (Benoit *et al*., 2006), and was not able to hydrolyse a tannase substrate, methyl gallate (Sharma *et al*., 2000). Overall, the substrate profile of AtFaeD was highly comparable with another characterized member of CE1 subfamily 5, AnFaeC (Dilokpimol *et al*., 2017). Although data on a smaller set of synthetic hydroxycinnamic acid substrates are available for another CE1 subfamily member, *A. sydowii* AsFaeC, this suggests that it possibly also has activity towards a broad substrate range, as it is active on methyl ferulate, methyl sinapate, methyl *p*‐coumarate and methyl caffeate (Dilokpimol *et al*., 2018).

**Figure 3:**
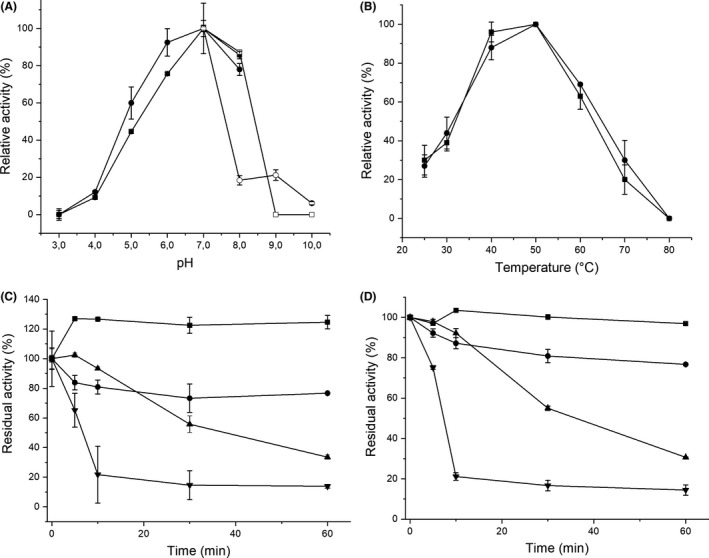
pH and temperature profiles, and thermostability of AtFaeD. (A) Residual activity of AtFaeD towards methyl 3,4‐dimethoxycinnamate (‐●‐) and methyl ferulate (‐■‐) in McIlvaine's buffer from pH 3.0 to 8.0, and towards methyl 3,4‐dimethoxycinnamate (‐○‐) and methyl ferulate (‐□‐) in glycine buffer from pH 7.0 to 10.0. (B) Residual activity of AtFaeD towards methyl 3,4‐dimethoxycinnamate (‐●‐) and methyl ferulate (‐■‐) from 25 to 80°C. Residual activity of AtFaeD towards (C) methyl 3,4‐dimethoxycinnamate and (D) methyl ferulate after 5 to 60 min incubation at 37°C (‐■‐), 45°C (‐●‐), 50°C (‐▲‐) and 60°C (‐▼‐). Vertical bars represent standard deviations of three replicate measurements.

AtFaeD showed a neutral pH optimum (Fig. 3A), similar to AnFaeC (Dilokpimol *et al*., 2017), AcFAE (Damásio *et al*., 2013) and ClFaeA1 (Kühnel *et al*., 2012), which all belong to CE1 subfamily 5 (Fig. S1) and/or to SF5 of fungal FAEs (Dilokpimol *et al*., 2016). The pH optimum of AtFaeD was higher than the pH 5.0 reported for the other *A. terreus* FAEs, AtFAE1/AtFaeA, AtFAE2 and AtFAE3, which are not members of family CE1 (Kumar *et al*., 2013; Zhang *et al*., 2015). AtFaeD was active from pH 4.0 to 8.0 towards methyl 3,4‐dimethoxycinnamate and methyl ferulate, and showed low activity towards methyl 3,4‐dimethoxycinnamate even at pH 9.0 and 10.0 (Fig. 3A).

AtFaeD was most active at 50°C, and 30% and 20% of activity was detected at 80°C towards methyl 3,4‐dimethoxycinnamate and methyl ferulate, respectively (Fig. 3B). More than 50% of the initial activity of AtFaeD was retained after 30 min incubation at 50°C, and around 80% and 15% of the residual activity was maintained after 60 min at 45°C and 60°C, respectively (Fig. 3C, D). This is in contrast with *A. terreus* AtFAE1/AtFaeA, AtFAE2 and AtFAE3, which are not stable above 40°C (Kumar *et al*., 2013). Also, a moderate increase (27%) in the activity towards methyl 3,4‐dimethoxycinnamate was observed after 5 to 60 min incubation at 37°C (Fig. 3C).

### *Aspergillus terreus* FaeD releases ferulic acid and xylooligosaccharides from insoluble plant biomass substrates

**Figure 4:**
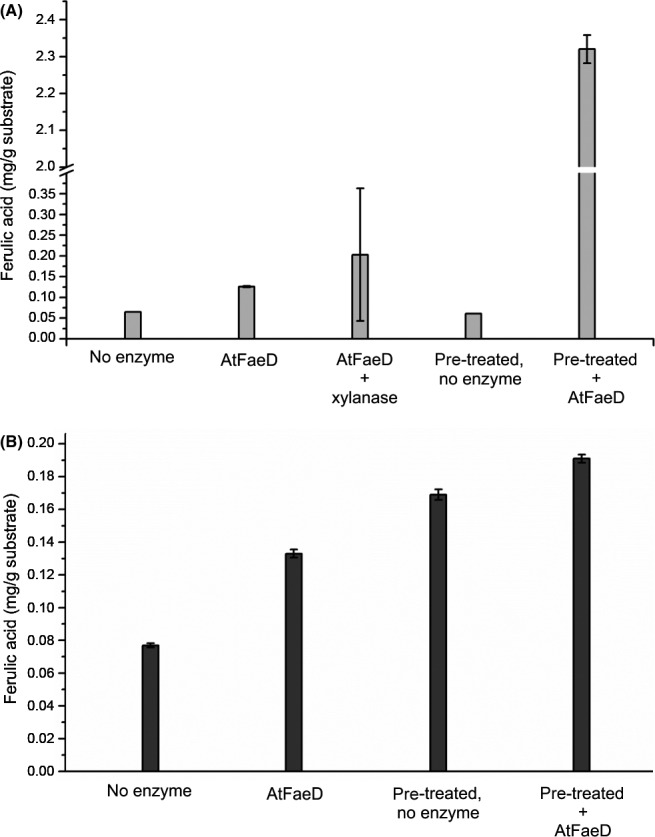
Release of ferulic acid from (A) 1% wheat arabinoxylan (WAX) and (B) 1% sugar beet pectin (SBP) using AtFaeD with and without pre‐treatment with commercial xylanase and endopolygalacturonase, respectively. WAX was also co‐incubated with AtFaeD and commercial xylanase. Mean values and standard deviations from three replicate experiments are presented.

**Figure 5:**
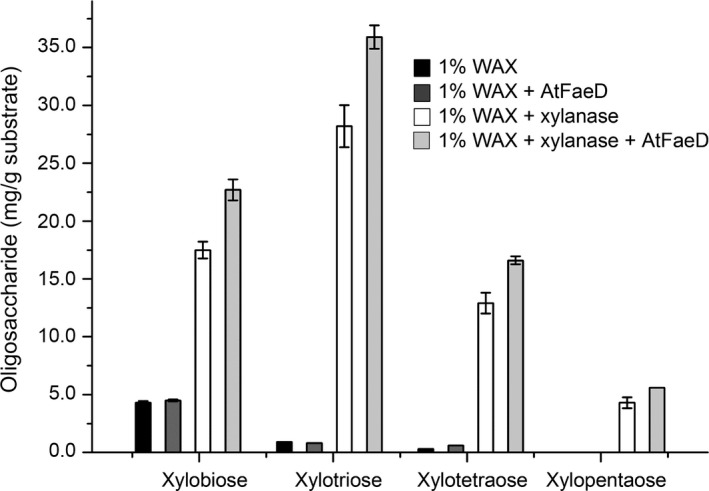
Oligosaccharide content of wheat arabinoxylan (WAX) before and after treatment with commercial xylanase and AtFaeD. Vertical error bars represent standard deviations of three replicate experiments.

In order to study the activity of AtFaeD on natural plant biomass substrates, the enzyme was tested for its ability to release ferulic acid and diferulic acids from insoluble WAX and SBP (Fig. 4), and xylooligosaccharides from WAX (Fig. 5). Whereas AtFaeD alone and in co‐incubation with a commercial xylanase slightly improved the yield of ferulic acid from WAX, it was shown to act sequentially with this xylanase by improving the release of ferulic acid from the pre‐treated WAX 11‐fold up to 2.3 mg g^−1^ (Fig. 4A). This is higher than the increase observed with AnFaeC, which has showed fourfold improvement in the release of ferulic acid from xylanase‐pre‐treated WAX (Dilokpimol *et al*., 2017). As WAX contains approximately 3 mg g^−1^ ferulic acid (analysed by The Complex Carbohydrate Research Center, CCRC), approximately 77% of ferulic acid was released when treated by co‐incubation of AtFaeD and xylanase. AtFAE1/AtFaeA, AtFAE2 and AtFAE3 from *A. terreus* have shown synergy with xylanase by improving ferulic acid release from de‐starched wheat and maize bran (Kumar *et al*., 2013), thus showing the ability of this fungus to produce a set of efficient FAE isoenzymes for plant biomass modification. SBP contains 1.9 mg g^−1^ ferulic acid, indicating that approximately 40% of ferulic acid was solubilized without enzymatic treatment (Fig. 4B). However, AtFaeD treatment increased the release of ferulic acid by 73%. Moreover, all ferulic acid was released from SBP when the endopolygalacturonase‐treated SBP was further hydrolysed by AtFaeD. Previously, no improvement in the release of ferulic acid was reported for AnFaeC incubated with endopolygalacturonase‐treated SBP (Dilokpimol *et al*., 2017). Also, pre‐treatment with debranching enzymes, such as endogalactanase, that have been successfully used with AnFaeA (de Vries *et al*., 2000) was suggested for AnFaeC (Dilokpimol *et al*., 2017). No release of diferulic acids by AtFaeD was detected. AtFaeD also acted synergistically with the commercial xylanase by improving the release of xylooligosaccharides from WAX by 27% to 30% (Fig. 5). This indicates that AtFaeD has potential in production of xylooligosaccharides, which are promising prebiotics that can be obtained from agricultural residues (Samanta *et al*., 2015).

## Experimental procedures

### Phylogenetic analysis of fungal CE1 proteins

Amino acid sequences for candidate fungal CE1 members were obtained from JGI Mycocosm (https://genome.jgi.doe.gov/programs/fungi/index.jsf) (Grigoriev *et al*., 2014) and through the CAZy database (http://www.cazy.org) (Lombard *et al*., 2014). The sequences were aligned using MAFFT (Multiple Alignment using Fast Fourier Transform) (Katoh *et al*., 2009). The alignment was manually checked, and genes with clearly erroneous gene models (e.g. with deletions, insertions or most likely incorrectly predicted start or stop codons or introns) were removed from the sequence set. In addition, recent gene duplications within a species with highly similar amino acid sequences (> 95% identity) were removed as they would affect the topology of the phylogenetic tree disproportionally. The amino acid sequence of *A. niger* FaeA and seven orthologs from the Eurotiomycetes order from FAE SF7 (Dilokpimol *et al*., 2016) were included in the set to be used as an outgroup in the phylogenetic analysis (Table S1). The alignment was re‐run with the modified sequence set and further analysed in MEGA7 (Kumar *et al*., 2016). Neighbour‐joining (NJ) (Saitou and Nei, 1987), minimal evolution (ME) and maximum likelihood (ML) trees were run, using 500 bootstraps (Felsenstein, 1985) to reveal the evolutionary relationship between the sequences. The final phylogenetic tree was based on the NJ tree, rooted to the FaeA branch and bootstrap values > 50 were indicated in the tree. For nodes that were also supported by bootstrap values > 50 in the ME and ML trees, the bootstrap values were indicated in the tree with the order: NJ, ME, ML.

### Bioinformatic analyses of *A. terreus* FaeD

The full‐length amino acid sequence of *A. terreus faeD* gene was obtained from JGI Mycocosm (protein ID 10207). SignalP 4.1 (http://www.cbs.dtu.dk/services/SignalP/, Petersen *et al*., 2011) was used for prediction of a putative signal peptide. The theoretical molecular mass and isoelectric point (p*I*) were calculated from the amino acid sequence without the signal peptide with ExPASy ProtParam tool (web.expasy.org/protparam/, Gasteiger *et al*., 2005). NetNGlyc 1.0 (http://www.cbs.dtu.dk/services/NetNGlyc; Gupta *et al*., in preparation) and NetOGlyc 4.0 (http://www.cbs.dtu.dk/services/NetOGlyc; Steentoft *et al*., 2013) were used for prediction of putative N‐ and O‐glycosylation sites, respectively.

### Microbial strains, isolation of *A. terreus* DNA, cloning and transformation of *faeD*

*Aspergillus terreus* CBS138435 (NIH2624) was obtained from the CBS Fungal Culture Collection (Utrecht, The Netherlands) and maintained on 2% (w/v) malt extract (ME, Biokar) agar plates. *Pichia pastoris* X33 (Invitrogen) was maintained on yeast extract‐peptone‐dextrose (YEPD) agar plates according to the manufacturer's instructions. *Escherichia coli* DH5α was used for bacterial transformation.

For DNA extraction, *A. terreus* was cultivated in 250 ml Erlenmeyer flasks in 50 ml 2% (w/v) ME liquid medium that was inoculated with 10^6^ spores ml^−1^ collected from 2% ME agar plates. The liquid cultures were incubated at 30°C and 250 rpm for 2 days, after which the mycelium was collected by filtering through Miracloth (Calbiochem). Genomic DNA was extracted from mycelium that was frozen in liquid nitrogen and ground with mortar and pestle using DNAzol (ThermoFisher) according to the instructions of the manufacturer.

The *A. terreus* FaeD (AtFaeD) encoding gene (accession no. EAU33188.1, 825 nt in length) was cloned downstream of the methanol‐inducible *AOX1* promoter region and α‐mating factor signal peptide from *Saccharomyces cerevisiae* in pPICZαA expression vector (Invitrogen). The expression vector contains C‐terminal peptides with c‐myc epitope and polyhistidine (6 × His) tag for detection and purification of recombinant protein. Genomic DNA was used for amplification of the *faeD* gene, which does not contain introns. Primers (sense: GAATGCGGCCGCGCAAACAGCGCTGGCTGC; antisense: GATGCGCTCTAGACCGAACTGAGAGAAAAAG TCCC, with NotI and XbaI restriction sites underlined, respectively) were designed according to the *faeD* gene sequence encoding the mature part of the corresponding amino acid sequence without the predicted N‐terminal signal peptide (Met1‐Ala21). The 50 μl PCR reaction mixture contained 80 ng of DNA template, 10 μl of 5 ×  HF buffer (ThermoFisher), 0.5 μM of sense and antisense primers, 1 μl of dNTP mixture (ThermoFisher), 1.5 μl of DMSO (100%) and 0.02 U of Phusion Hot Start DNA polymerase (ThermoFisher). PCR was performed with initial denaturation at 98°C for 30 s, 40 cycles of denaturation at 98°C for 10 s, annealing at 55°C for 30 s, elongation at 72°C for 20 s and final extension at 72°C for 5 min. The PCR product was run on a 1% agarose gel and extracted using Gene JetTM Gel Extraction kit (ThermoFisher). After restriction digest of the PCR fragment, an expression vector was constructed by ligating (T4 DNA ligase, ThermoFisher) the amplified product into pPICZαA expression vector downstream of *Saccharomyces cerevisiae* α‐mating factor signal sequence. The expression vector was transformed into *E. coli* DH5α cells and sequenced (Macrogen, The Netherlands) to verify the correct reading frame of *faeD*.

The construct was linearized with SacI and electroporated to *P. pastoris* X33‐competent cells according to the instructions of the manufacturer (BioRad GenePulser, BioRad). YEPD agar plates with 100 μg ml^−1^ zeocin (Invitrogen) were used for selection of transformants.

### Production of recombinant *A. terreus* FaeD in *P. pastoris* by fed‐batch fermentation

*Aspergillus terreus* FaeD was produced in fed‐batch fermentation in a 5 L stirred tank bioreactor (Biostat B5, Sartorius) operated with BioPAT^®^ MFCS software using the parameters for methanol feeding of *P. pastoris* Mut^+^ strains according to the instructions of the manufacturer (Invitrogen). For inoculum, yeast cells were cultivated in five 250 ml Erlenmeyer flasks containing 50 ml of yeast extract‐peptone medium supplemented with 1% glycerol (YEPG) at 30°C, 250 rpm, overnight. The cells were harvested by centrifugation (1500 × *g*, 10 min, 20°C) and suspended in 100 ml of basal salts (BS) medium prior inoculation of the bioreactor. The volume of the inoculum corresponded to 10% (v/v) of the fermentation starting volume (2.5 L). One litre of the BS medium contained 0.93 g CaSO_4_, 35 g glycerol, 26.7 ml 85% H_3_PO_4_, 4.13 g KOH, 18.2 g K_2_SO_4_ and 14.9 g MgSO_4_, and was supplemented with 4.35 ml *Pichia* trace metals (PTM1) solution that contained 0.2 g l^−1^ biotin, 0.5 g l^−1^ CoCl_2_, 6 g l^−1^ CuSO_4_·5H_2_O, 65 g l^−1^ FeSO_4_·7H_2_O, 0.02 g l^−1^ H_3_BO_3_, 5 ml l^−1^ H_2_SO_4_, 3.0 g l^−1^ MnSO_4_·H_2_O, 0.08  g l^−1^ NaI, 0.2 g l^−1^ Na_2_MoO_24_·2H_2_O and 20 g l^−1^ ZnCl_2_.

Fermentation was started with a batch phase with 35 g l^−1^ glycerol in an initial volume of 2.5 L of BS medium. The fermentation was performed at 30°C, under cascade‐controlled agitation with aeration of 2.5 vessel volumes/min (vvm) to keep 20% saturation of dissolved oxygen. The pH of the medium was automatically maintained pH 5.0 with 25% (v/v) NH_4_OH. After the initial glycerol was consumed, a fed‐batch phase with a feeding of 87% glycerol was started and continued for 4 h. This was followed by an induction phase, during which the production of recombinant AtFaeD was induced by feeding 100% methanol into the culture medium for 70 h. A volume of 12 ml l^−1^ PTM1 was fed into the bioreactor as a part of the glycerol and methanol feeds. Both the glycerol and methanol feed rates were manually adjusted in order to reach a substrate‐limited cultivation. Struktol (Schill Seilacher) was used to reduce foaming. The bioreactor was sampled every 20 h to monitor protein concentration and FAE activity. After fermentation, supernatant was collected by centrifugation (3000 × *g*, 4°C) and concentrated with a tangential flow filtration (FiltronMinisette apparatus, 10 000 NMWL filter cassettes) and Amicon ultrafiltration unit (Millipore) with 10 000 NMWL polyethersulphone membrane (Sartorius, Germany) at 4°C.

### Biochemical properties of *A. terreus* FaeD

The molecular mass of AtFaeD was estimated on SDS‐PAGE in 12% Criterion™ XT Bis‐Tris gels (Bio‐Rad) under denaturing conditions with 5% β‐mercaptoethanol and extended boiling of the sample (20 min). PageRuler Prestained Protein Ladder was used as a molecular mass marker (ThermoFisher) and the proteins were visualized with PageBlue™ protein staining solution (ThermoFisher). For deglycosylation, 6 μl of concentrated *P. pastoris* culture supernatant was treated with peptide‐N‐glycosidase F (PNGase F), Endoglycosidase H_f_ (Endo H_f_) and endo‐α‐N‐acetylgalactosaminidase (*O*‐glycosidase) according to the instructions of the manufacturer (New England Biolabs). SDS‐PAGE separated AtFaeD was electroblotted to nitrocellulose membrane (Optitran BA‐S 83 reinforced Nitrocellulose, Schleicher & Schuell). Anti‐His(C‐term) antibody (Invitrogen) was used as a primary and anti‐mouse IgG linked to alkaline phosphatase (cell signalling technology) as a secondary antibody according to the instructions of the manufacturers. Visualization of proteins was performed with a BCIP/NBT colorimetric assay according to the instructions of the manufacturer (Bio‐Rad).

The carbohydrate content of AtFaeD was analysed using phenol‐sulphuric acid assay (Masuko *et al*., 2005) using d‐glucose (Sigma‐Aldrich) and d‐mannose (Sigma‐Aldrich) as standards.

### Determination of enzyme activities and protein concentration

The enzymatic activity of AtFaeD was determined spectrophotometrically (UV‐Vis‐1700 PharmaSpec, Shimadzu) against different synthetic methyl and ethyl esters as well as chlorogenic acid (Apin Chemicals, Ltd.) and methyl gallate (Sigma‐Aldrich). Change of absorbance was monitored at 255 nm for methyl 4‐hydroxybenzoate (ε_255_ = 14 300 M^−1^ cm^−1^), 297 nm for methyl cinnamate (ε_297_ = 18 310 M^−1^ cm^−1^), 308 nm for methyl *p*‐coumarate (ε_308_ = 20 390 M^−1^ cm^−1^) and ethyl *p‐*coumarate (ε_308_ = 17 980 M^−1^ cm^−1^), 320 nm for methyl 3,4‐dimethoxycinnamate (ε_320_ = 10 670 M^−1^ cm^−1^), methyl ferulate (ε_320_ = 29 680 M^−1^ cm^−1^) and methyl sinapate (ε_320_ = 15 890 M^−1^ cm^−1^), 322 nm for methyl caffeate (ε_322_ = 14 720 M^−1^ cm^−1^) and ethyl ferulate (ε_322_ = 15 950 M^−1 ^cm^−1^), and 323 nm for chlorogenic acid (ε_323_ = 16 860 M^−1 ^cm^−1^) at 37°C in 100 mM MOPS buffer, pH 6.0, according to Benoit *et al*. (2015). Tannase activity towards methyl gallate was determined at 30°C in 0.1 M sodium phosphate buffer, pH 6.0, by detecting the formation of the chromogen between gallic acid and rhodanine (Fluka) according to Sharma *et al*. (2000). The amount of formed gallic acid was determined from the standard curve with 1.0–100 μg ml^−1^ gallic acid. All assays were performed in triplicate.

Temperature optimum and thermostability of AtFaeD were deduced from the residual activity towards methyl 3,4‐dimethoxycinnamate and methyl ferulate. The temperature optimum was determined from 25°C to 80°C using similar conditions as described above. For thermostability, the enzyme was incubated for 5, 10, 30 and 60 min at temperatures from 37°C to 60°C in 100 mM MOPS buffer, pH 6.0, after which the residual activity was assayed at 37°C as described earlier. The pH profile was determined at 37°C from pH 3.0 to 8.0 in McIlvaine's buffer (McIlvaine, 1921) and from pH 7.0 to 10.0 in 0.2 M glycine buffer using methyl 3,4‐dimethoxycinnamate and methyl ferulate as substrates under similar conditions as described earlier. Three technical replicate reactions were used in all the measurements.

Protein concentration was determined using PierceTM BCA protein assay reagent kit according to the instructions of the manufacturer (ThermoFisher).

### Hydrolytic activity of *A. ferreus* FaeD towards polysaccharides

*Aspergillus terreus* FaeD activity was determined towards insoluble wheat arabinoxylan (WAX; Megazyme) and sugar beet pectin (SBP; Pectin Betapec RU301, Herbstreith & Fox KG). As described earlier, 1% of WAX or SBP was prepared in 50 mM sodium acetate buffer, pH 4.5 (de Vries *et al*., 2000). A reaction mixture containing 500 μl 1% substrate and 100 μl enzyme concentrate (approx. 10 μg AtFaeD) was performed in 2 ml Eppendorf tube and incubated at 30°C, 100 rpm, for 24 h. With WAX, AtFaeD was also co‐incubated with 1 μg xylanase (from *Thermomyces lanuginosus*, Sigma Aldrich). The enzymes were inactivated by heating at 100°C for 10 min. In case of pre‐treatment, the substrate mixtures were incubated with 0.1 mg xylanase for WAX or 0.1 mg endopolygalacturonase (pectinase from *A. niger*, Sigma Aldrich) for SBP, at 30°C, 100 rpm for 72 h, followed by heat inactivation at 100°C for 10 min prior to incubation with AtFaeD. Reactions without the enzymes were conducted as controls to take into account the possible effect of heating to the release of hydroxycinnamic acids from the plant biomass substrates.

### Release of hydroxycinnamic acids from wheat arabinoxylan and sugar beet pectin

Release of ferulic and *p*‐coumaric acid, and 8,5′‐, 5,5′‐ and 8‐*O*‐4‐diferulic acids from the enzyme‐treated WAX and SBP was monitored by HPLC (Dionex ICS‐5000 + chromatography system; Thermo Scientific) equipped with an Acclaim Mixed‐Mode WAX‐1 LC Column (3 × 150 mm; Thermo Scientific) and a UV detector (310 nm; Thermo Scientific). The chromatographic separation was performed according to Dilokpimol *et al*. (2017). Ferulic acid (0.25–50 mM; Acros) was used as a standard for identification and quantitation; 8,5′, 5,5′ and 8‐*O*‐4 ‐diferulic acids were synthesized as described previously (Dilokpimol *et al*., 2017), and were used to identify potentially liberated diferulic acids. The amount of the hydroxycinnamic acids detected in the non‐enzyme‐treated control samples were subtracted from the enzyme‐treated samples.

### Release of xylooligosaccharides from wheat arabinoxylan

Xylooligosaccharides released from WAX before and after co‐incubation with AtFaeD and commercial xylanase were analysed from peak areas in HPAEC‐PAD (Dionex ICS‐5000 + system; Thermo Scientific, Sunnyvale, CA) equipped with CarboPac PA1 column (2 × 250 mm with 2 × 50 mm guard column; Thermo Scientific) using a linear gradient of 0‐400 mM sodium acetate in 100 mM NaOH for 45 min, followed by an isocratic elution of 900 mM sodium acetate in 100 mM NaOH for 10 min (20°C, flow rate: 0.30 ml min^−1^). The column was cleaned up with 1 M NaOH for 5 min followed by 10 min equilibration using 100 mM NaOH. Five to 200 μM xylobiose, xylotriose, xylotetraose and xylopentaose (Megazyme) were used as standards.

## Conclusions

Fungal members of CAZy family CE1 encode enzymes with different esterase activities. In this study, we were able to divide the family CE1 into five subfamilies and assign putative functions to the members of three of these subfamilies. This enables faster function assignment of CE1 enzymes from fungal genomes, which facilitates gene selection for characterization and application in biotechnological processes. In addition, heterologously produced *A. terreus* FaeD from CE1 subfamily 5 confirmed the conservation of substrate specificity within such a subfamily by showing a broad specificity towards synthetic methyl and ethyl esters as well as neutral pH optimum. AtFaeD also released ferulic acid from plant biomass substrates, further confirming its true FAE activity. This result was similar to what was previously reported for another member of this subfamily, *A. niger* FaeC, but AtFaeD appears to benefit more from pre‐incubation of WAX with xylanase than AnFaeC. Together, these characteristics make AtFaeD a potential candidate for biotechnological applications.

## Conflict of interest

The authors declare that they have no conflict of interests.

## Supporting information


**Table S1**. Amino acid sequences used for the CE1 phylogeny.

**Fig. S1**. Phylogenetic tree of fungal CE1 genes based on amino acid sequences.

**Fig. S2**. (A) Total protein concentration (‐■‐) and activity towards methyl ferulate (‐●‐) followed from the extracellular culture liquid of *P. pastoris* during fermentation of recombinant AtFaeD. Vertical bars represent standard deviations of three technical replicates in BCA assay. (B) SDS‐PAGE of AtFaeD with and without treatment with Endo H_f_, PNGase and *O*‐glycosidase enzymes. The glycosidases are marked with arrows. (C) Immunodetection of AtFaeD with anti‐His antibody.
