# Supplementary material for: Characterization of a feruloyl esterase from Aspergillus terreus facilitates the division of fungal enzymes from Carbohydrate Esterase family 1 of the carbohydrate‐active enzymes (CAZy) database
Source: Microb Biotechnol. 2018 Apr 26;11(5):869–80. doi: 10.1111/1751-7915.13273 (PMC6116738; doi:10.1111/1751-7915.13273)
Supplement: Supplementary file 2 — Fig. S1. Phylogenetic tree of fungal CE1 genes based on amino acid sequences. [file MBT2-11-869-s002.pdf]

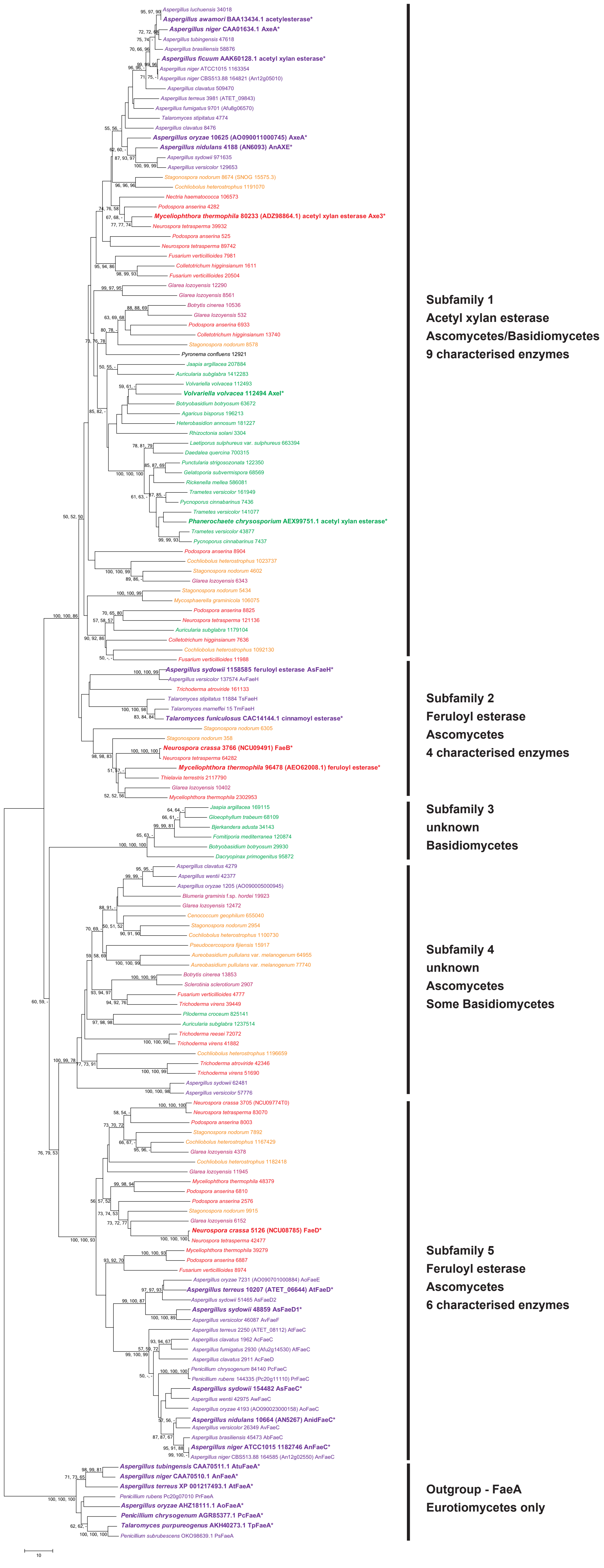

Supporting Figure 1. Phylogenetic tree of fungal CE1 genes based on amino acid sequences.
The evolutionary history was inferred using the Neighbor-Joining (NJ) method [1] and the optimal tree is shown. Bootstrap values (500 replicates) are shown next to the branches [2], also of a Minimal Evolution (ME) and Maximum Likelihood (ML) tree using the same dataset (order: NJ, ME, ML). Only bootstrap values >50 are shown. The tree is drawn to scale, with branch lengths in the same units as those of the evolutionary distances used to infer the phylogenetic tree. Evolutionary analyses were conducted in MEGA7 [3]. The FAE subfamily 7 [4] was used as an outgroup. Species were color-coded according to their taxonomic clade: Blue = Eurotiomycetes, Orange = Dothidiomycetes, Red = Sordariomycetes, Purple = Leotiomycetes, Black = Pezizomycetes, Green = Agaricomycotina. Functionally characterised enzymes are marked with an * and in boldface and larger font.

1.Saitou N, Nei M. 1987. The neighbor-joining method: A new method for reconstructing phylogenetic trees. Mol Biol Evol 4:406-425.
2.Felsenstein J. 1985. Confidence limits on phylogenies: An approach using the bootstrap. Evolution 39:783-791.
3.Kumar S, Stecher G, Tamura K. 2016. MEGA7: Molecular Evolutionary Genetics Analysis version 7.0 for bigger datasets. Mol Biol Evol 33:1870-1874.
4.Dilokpimol A, Mäkelä MR, Aguilar-Pontes MV, Benoit-Gelber I, Hilden KS, de Vries RP. 2016. Diversity of fungal feruloyl esterases: updated phylogenetic classification, propertie-sand industrial applications. Biotechnol Biofuels 9: 1-18.
