# Supplementary material for: Characterization of a feruloyl esterase from Aspergillus terreus facilitates the division of fungal enzymes from Carbohydrate Esterase family 1 of the carbohydrate‐active enzymes (CAZy) database
Source: Microb Biotechnol. 2018 Apr 26;11(5):869–80. doi: 10.1111/1751-7915.13273 (PMC6116738; doi:10.1111/1751-7915.13273)
Supplement: Supplementary file 3 — Fig. S2. (A) Total protein concentration (‐■‐) and activity towards methyl ferulate (‐●‐) followed from the extracellular culture liquid of P. pastoris during fermentation of recombinant AtFaeD. Vertical bars represent standard deviations of three technical replicates in BCA assay. (B) SDS‐PAGE of AtFaeD with and without treatment with Endo Hf, PNGase and O‐glycosidase enzymes. The glycosidases are marked with arrows. (C) Immunodetection of AtFaeD with anti‐His antibody. [file MBT2-11-869-s003.pdf]

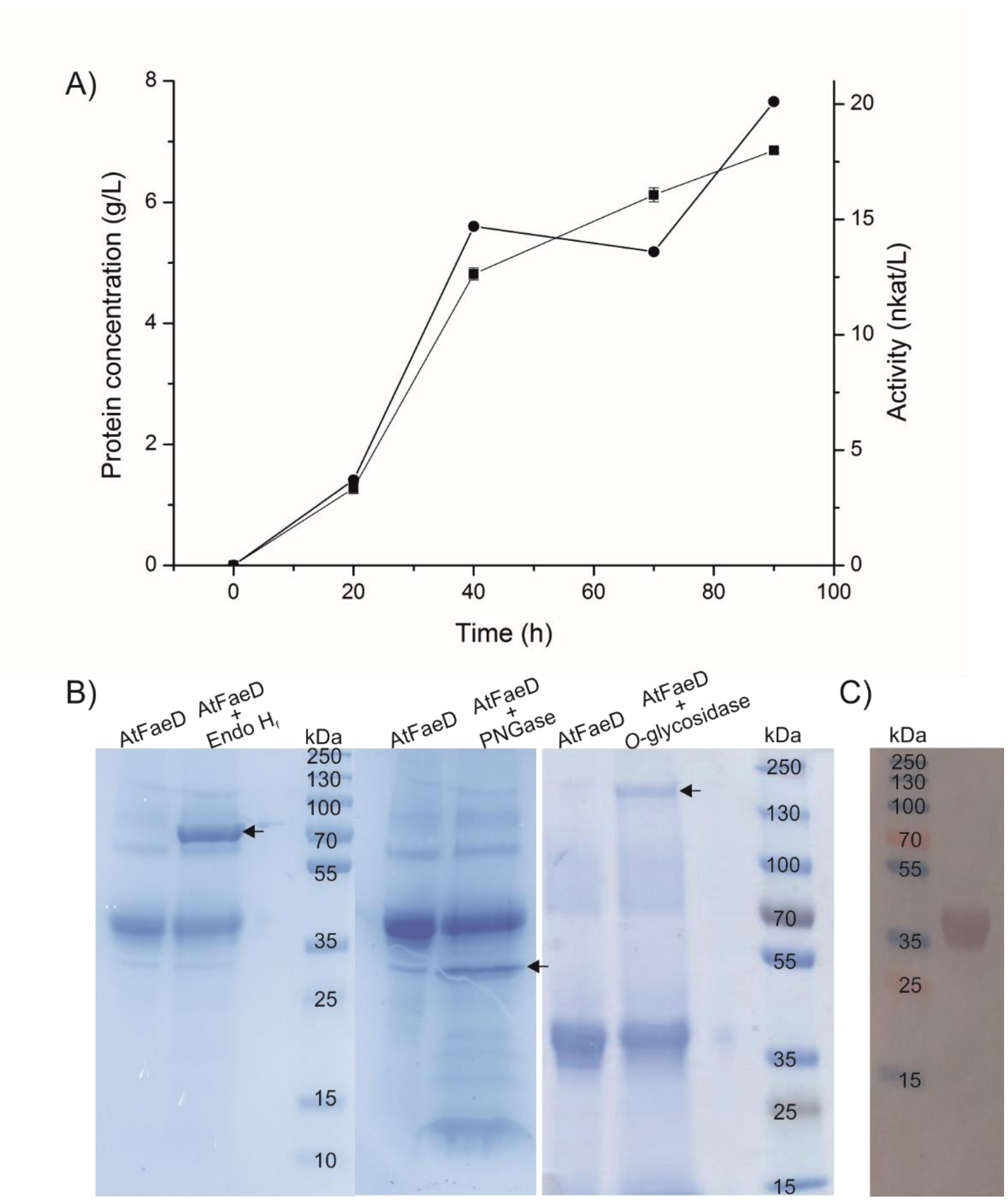

Supporting Figure 2. A) Total protein concentration (-■-) and activity towards methyl ferulate (-●-) followed from the extracellular culture liquid of *P. pastoris* during fermentation of recombinant AtFaeD. Vertical bars represent standard deviation of three technical replicates in BCA assay. B) SDS-PAGE of AtFaeD with and without treatment with Endo H_f, PNGase and *O*-glycosidase enzymes. The glycosidases are marked with arrows. C) Immunodetection of AtFaeD with anti-His antibody.
